# mPGES-1 Inhibitor Discovery Based on Computer-Aided Screening: Pharmacophore Models, Molecular Docking, ADMET, and MD Simulations

**DOI:** 10.3390/molecules28166059

**Published:** 2023-08-15

**Authors:** Qiqi Huang, Tianli Lai, Qu Wang, Lianxiang Luo

**Affiliations:** 1 The First Clinical College, Guangdong Medical University, Zhanjiang 524023, China; hqq77@gdmu.edu.cn (Q.H.); laitianli@gdmu.edu.cn (T.L.); 2 The Marine Biomedical Research Institute, Guangdong Medical University, Zhanjiang 524023, China; wangqu0503@gdmu.edu; 3 The Marine Biomedical Research Institute of Guangdong Zhanjiang, Zhanjiang 524023, China

**Keywords:** mPGES-1, pharmacophore modeling, molecular docking, ADMET, MD simulation

## Abstract

mPGES-1 is an enzyme, which, when activated by inflammatory factors, can cause prostaglandin E synthesis. Traditional non-steroidal anti-inflammatory drugs are capable of inhibiting prostaglandin production, yet they can also cause gastrointestinal reactions and coagulation disorders. mPGES-1, the enzyme at the conclusion of prostaglandin production, does not cause any adverse reactions when inhibited. Numerous studies have demonstrated that mPGES-1 is more abundant in cancerous cells than in healthy cells, indicating that decreasing the expression of mPGES-1 could be a potential therapeutic strategy for cancer. Consequently, the invention of mPGES-1 inhibitors presents a fresh avenue for the treatment of inflammation and cancer. Incorporating a database of TCM compounds, we collected a batch of compounds that had an inhibitory effect on mPGES-1 and possessed IC50 value. Firstly, a pharmacophore model was constructed, and the TCM database was screened, and the compounds with score cut-off values of more than 1 were retained. Then, the compounds retained after being screened via the pharmacodynamic model were screened for docking at the mPGES-1 binding site, followed by high-throughput virtual screening [HTVS] and standard precision [SP] and super-precision [XP] docking, and the compounds in the top 20% of the XP docking score were selected to calculate the total free binding energy of MM-GBSA. The best ten compounds were chosen by comparing their score against the reference ligand 4U9 and the MM-GBSA_dG_Bind score. ADMET analysis resulted in the selection of ten compounds, three of which had desirable medicinal properties. Finally, the binding energy of the target protein mPGES-1 and the candidate ligand compound was analyzed using a 100 ns molecular dynamics simulation of the reference ligand 4U9 and three selected compounds. After a gradual screening study and analysis, we identified a structure that is superior to the reference ligand 4U9 in all aspects, namely compound 15643. Taken together, the results of this study reveal a structure that can be used to inhibit mPGES-1 compound 15643, thereby providing a new option for anti-inflammatory and anti-tumor drugs.

## 1. Introduction

Prostaglandin PG plays an important role in mediating cell proliferation, differentiation, and apoptosis after binding with specific receptors. In addition, prostaglandins are also involved in the pathological processes of inflammation, cancer, and various cardiovascular diseases [1]. COX is a key enzyme that mediates the conversion of arachidonic acid into prostaglandin. Traditional non-steroidal anti-inflammatory drugs (NSAIDs) produce anti-inflammatory effects by directly inhibiting COX and blocking the production of prostaglandins. However, direct blocking of COX can cause gastrointestinal mucosal damage and coagulation dysfunction [2]. In order to avoid the side effects of NSAIDs, this study focused on microsomal prostaglandin E synthase-1 (mPGES-1), the terminal enzyme downstream of COX-2 in inducible PGE [3]. mPGES-1 is low in normal tissue and is induced in any inflammation. Studies have shown that it is overexpressed in a variety of human cancers, such as lung cancer [4], stomach cancer [5], colon cancer [6], prostate cancer [7], breast cancer [8], cervical cancer [9], pancreatic cancer [10], melanoma [11], squamous carcinoma of the head and neck [12], papillary carcinoma of the thyroid [13], and glioma [14]. Therefore, mPGES-1 has become an important target for the treatment of acute and chronic inflammatory diseases and cancers as shown in Supplementary Figure S1 [15].

Inhibition of mPGES-1 for playing an anti-tumor and anti-inflammatory role has attracted much attention from researchers. Several mPGES-1 inhibitors have been identified in the past; however, there are currently no mPGES-1 inhibitors on the market. Therefore, the discovery of novel mPGES-1 inhibitors for the treatment of cancer and inflammation provokes our thinking and research [16]. In addition, in recent years, the research of traditional Chinese medicine has been deepening day by day. Traditional Chinese medicine will greatly support its modernization and guide rational modern drug discovery. It has been proven that traditional Chinese medicine plays an important role in inhibiting inflammation and tumors [17,18]. In recent years, with the development and application of Chinese herbal medicine, various Chinese herbal preparations have continuously been discovered and applied in clinical practice. We collected the structural information of small molecules from traditional Chinese medicines that can be found on the internet and integrated it to carry out virtual drug screening for mPGES-1 targets. In this study, we hope to screen out mPGES-1 inhibitors from the traditional Chinese medicine database and provide new choices for the development of anti-inflammatory and anti-tumor drugs.

Computer-aided drug design has been widely used in the discovery of lead compounds [19,20]. This work has greatly accelerated the speed of drug design and has guided researchers to better develop new drugs on the basis of science [21]. In this study, a ligand-based and structure-based approach to drug design was used: pharmacophore models, molecular docking screening, ADMET, and kinetic simulations were used to identify novel compounds as potential mPGES-1 inhibitors. After study and analysis, we determined a compound with better scores and stability than the reference ligand. The specific workflow is shown in Supplementary Figure S2.

## 2. Results

### 2.1. Pharmacophore Model Establishment

**Figure 1:**
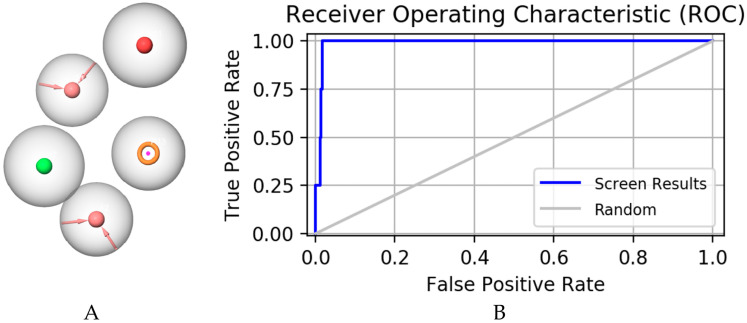
Pharmacophore model constructed as shown in (**A**): a hydrogen bond donor, two hydrogen bond acceptors, an aromatic ring, and a negatively charged ion center; (**B**) represents receiver operating characteristic curve (ROC) of pharmacophore model.

Pharmacophore comprises the physical and chemical characteristics and spatial arrangement necessary for the molecular recognition of ligands by biomacromolecules. Bioactive compounds with specific targets or similar bioactive compounds can be obtained via a screening compounds database with a pharmacophore model [22]. Pharmacophore models can be structure-based or ligand-based. In this study, a series of three-dimensional pharmacophore models of mPGES-1 were constructed based on the reported inhibitors, and the common characteristics of biological activity were analyzed by superimposing these inhibitory compounds. Supplementary Figure S3 shows the collected compounds. The best pharmacophore model (AAHNR) consists of five features: a hydrogen bond donor, two hydrogen bond acceptors, an aromatic ring, and a negatively charged ion center, as shown in Figure 1.

### 2.2. Pharmacophore Model Verification

**Figure 2:**
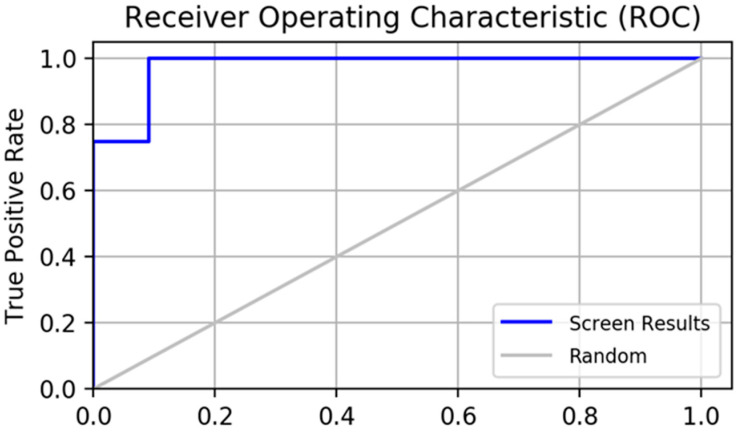
The receiver operating characteristic curve (ROC) of the pharmacophore constructed by this institute validates its results. The full name of ROC is receiver operating characteristic curve, and the abscissa of this curve is the false positive rate (FPR). The ordinate is the true positive rate (TPR).

**Figure 3:**
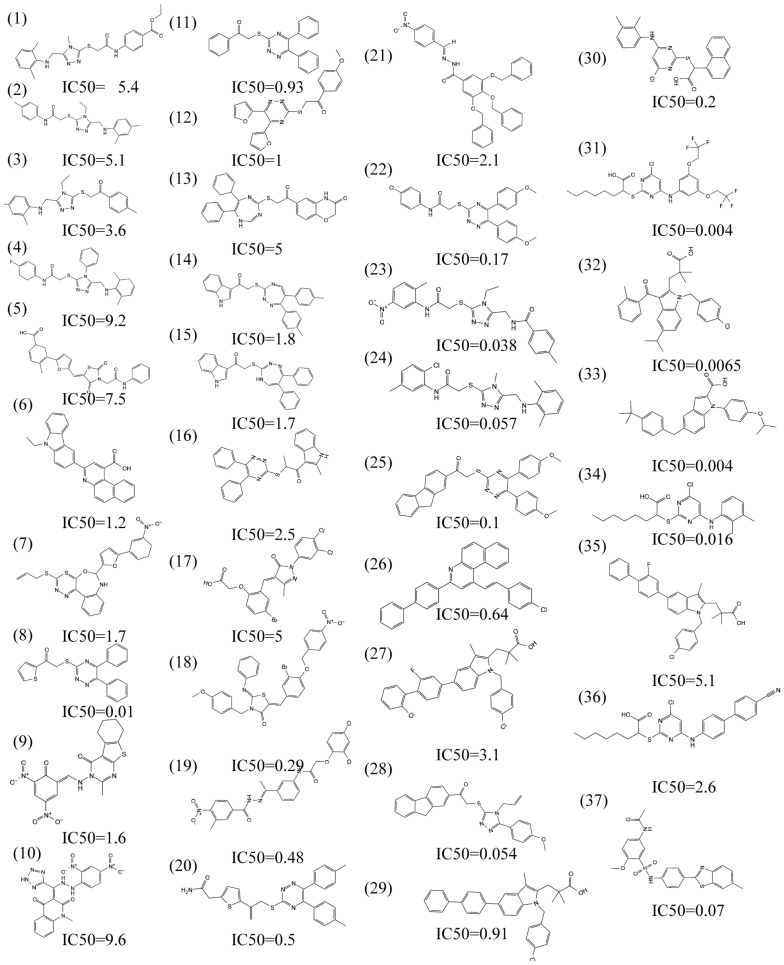
The virtual screening workflow (VSW) used in this study was used to identify targeted mPGES-1 inhibitors.

A pharmacophore model for screening should have the ability to accurately distinguish between active and inactive compounds [23]. The receiver operating characteristic curve (ROC), which is characterized by a false positive rate (FPR) as the abscissa and a true positive rate (TPR) as the ordinate. The closer the *X*-axis is to zero, the higher the accuracy is. The larger the *Y*-axis, the better the accuracy. The whole figure is divided into two parts according to the position of the curve. The area under the curve is called the AUC (area under the curve) and is used to indicate the accuracy of the forecast [24]. The higher the AUC value, the larger the area under the curve, indicating a higher prediction accuracy. The closer the curve is to the upper left corner (the smaller the X, the larger the Y), the higher the prediction accuracy [25]. In this study, the AUC (area under ROC curve is 0.77, as shown in Figure 2) was verified using this model, which has a good ability to distinguish active compounds from decoy compounds. Figure 3 shows the compounds used to build the verification set.

### 2.3. Molecular Docking

**Figure 4:**
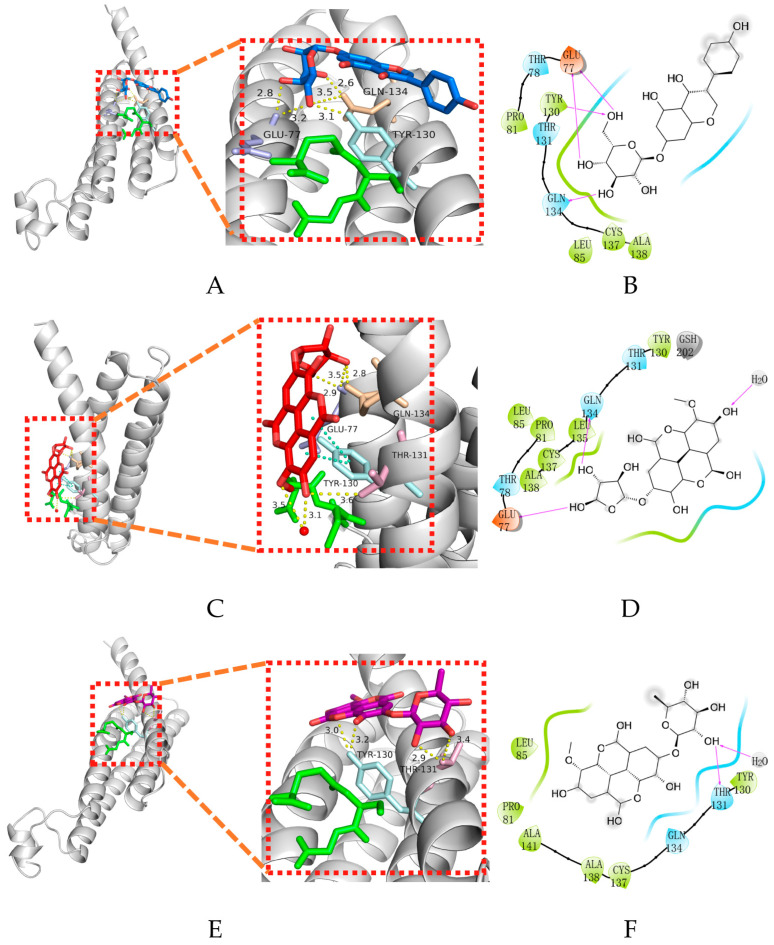
Interactions between protein–ligand complexes. (**A**) Three-dimensional binding pattern of protein and compound 14186; (**B**) two-dimensional binding pattern of protein and compound 14186; (**C**) three-dimensional binding pattern of protein and compound 14294; (**D**) two-dimensional binding pattern of protein and compound 14294; (**E**) three-dimensional binding pattern of protein and compound 15643; and (**F**) two-dimensional binding pattern of protein and compound 15643.

Molecular docking is an important part of the drug design process, which is used to identify the bioactive conformation of small and medium molecules at protein binding sites and to analyze the interactions between protein ligands [26]. This study evaluated the binding capacity of compounds important to the mPGES-1 protein. Glide is a virtual screening process from HTVS to SP to XP that further eliminates false positives with more extensive sampling and advanced scoring, resulting in higher enrichment. The Glide module processes compounds by searching for the conformation, orientation, and spatial position of the docking ligand. Firstly, the search space is reduced via rough positioning and scoring, and then the energy optimization of the candidate posture is carried out using the OPLS–AA non-bonding potential grid. Finally, the optimal docking posture is selected and evaluated using the function model combining experience and force field [26]. In this study, receptor grids with X = 9.62, Y = 20.16, and Z = 12.64 were prepared using the Glide module based on known binding sites. The Maestro software was used to dock the compounds with mPGES-1 to analyze and evaluate their binding ability. Table 1 shows the comparison of 10 compounds with 4U9 with better butt scores and MMGBSA scores than the reference ligand 4U9. The binding affinities of compound 14294, compound 15643, and compound 14186 were −5.558 kcal/mol, −4.664 kcal/mol, and −5.202 kcal/mol, respectively. Their binding affinity is better than that of the inhibitor (−4.605 kcal/mol) in the protein ligand complex (PDB ID: 4YL3) (https://www.rcsb.org/, accessed on 2 May 2022). The interaction of compound 14294 in the docking complex is shown in Figure 4A,B. The interaction of compound 15643 is shown in Figure 4C,D. The interaction of compound 14186 is shown in Figure 4E,F. Compound 14294 can be observed to form hydrogen bonds with GLN134, GLU77, and THR131. The Pi–Pi interaction established with TYR130 also plays a key role in ligand–receptor binding. Compound 15643 forms hydrogen bonds with TYR130 and THR131. In addition, compound 14186 forms hydrogen bond interactions with GLN134, TYR130, and GLU77. Analysis showed that the rich interaction types between compound 14294 and the mPGES-1 protein resulted in the best docking results. It can be seen from the interaction analysis that the docking results are reliable, and the selected compounds can be further analyzed.

### 2.4. ADMET Analysis

Absorption, distribution, metabolism, elimination, and toxicity (ADMET) characteristics are important in determining the efficacy and safety of drug candidates [27]. It is important to predict ADMET in order to avoid drug failure in late clinical trials. After excluding three false positive structures using “False Positive Remover”, drug absorption, distribution, metabolism, excretion, and toxicity were tested using “SwissADME”. A total of 17 related indexes were analyzed, including lipophilicity, hydrogen bond, solubility, permeability, etc., and they were compared with positive control compounds; finally, three compounds were obtained that were more similar to drug-like molecules [28]. The results of ADMET analysis of the compounds are shown in Table 2.

The logS value reflects the solubility of the drug. The smaller the value, the less soluble the compound is in water. When LogS < −6.0, the compound is almost insoluble in water. As shown in Table 2, the reference compound 4U9LogS < −6 is difficult to dissolve in water, while the selected compounds have better water solubility, which is related to their higher number of hydrogen bond receptors and hydrogen bond donors. The more hydrogen bond interactions, the better the hydrophilicity of the compound; the more polar interactions formed by Pi, the better the lipophilicity. The LogP value is the logarithmic value of the ratio of the partition coefficient of the compound in n-octanol and water, indicating the oil–water partition coefficient of the substance. The reference ligand 4U9 has a higher lipophilicity, while the screened compound has a lower lipophilicity. In addition, compound 4U9 and the three candidate compounds all had low gastrointestinal absorption and high skin penetration. BBB permeation is used to assess the ability of chemicals to cross the blood–brain barrier, which is a must for central drugs. The mPGES-1 inhibitors developed in this study belong to the non-central class of inhibitors, and none of the four compounds cross the blood–brain barrier. P-glycoprotein is related to the availability of compounds in the body. Compound 4U9 is P-glycoprotein substrate, so it is more likely to be pumped out of cells by P-glycoprotein in an ATP-dependent transport mode. The three candidate compounds are non-P-glycoprotein substrates and have better bioavailability. Using comprehensive analysis, compound 15643, compound 14186, and compound 14294 had higher bioavailability scores than compound 4U9. 

In terms of toxicity prediction, six important toxicity assessments of Hepatotoxicity, Carcinogenicity, Immunotoxicity, Mutagenicity, Cytotoxicity, and LD50 were carried out in this study. The predicted toxicity class of compound 15643 and compound 14186 is 5, which is higher than compound 4U9.

### 2.5. MD Simulation

MD simulations are used to study the binding stability of small molecules and target proteins. RMSD and RMSF analysis, hydrogen bond analysis, and MM-PBSA analysis were performed on the selected small molecules. The analysis results are presented below.

#### 2.5.1. RMSD and RMSF Analysis

**Figure 5:**
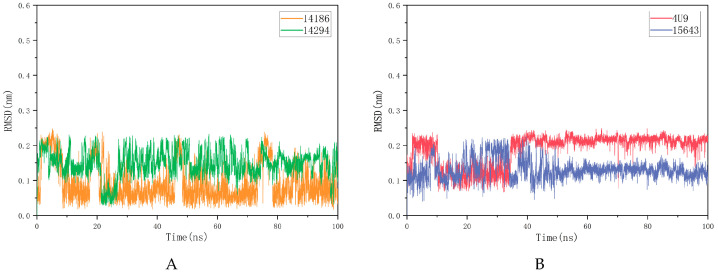
Compound 4U9 and three candidate compounds are plotted as root mean square deviation (RMSD) after fitting to the protein, respectively. (**A**) shows RMSDs of 4U9 and compound 15643 for 100 ns after fitting to mPGES-1, respectively. (**B**) shows RMSDs of compound 14186 and 14294 after fitting with mPGES-1 for 100 ns, respectively.

The RMSD value reflects the degree to which the atoms deviate from the average position, that is, the size of the motion of each atom. The molecular RMSD is shown in Figure 5A,B. Compound 15643 showed no significant difference in RMSD values at the beginning and end of MD, but significant fluctuations were observed over a period of 7–49 ns before stabilizing. Compound 14186 showed several distinct spikes in 100 ns MD but eventually stabilized. Compound 14294 fluctuated in 100 ns MD and was ultimately not completely stable. Although the stability of RMSD is different, all three molecules have low average RMSD. The average RMSDs of 15643, 14186 and 14294 were 0.1306 nm, 0.0925 nm and 0.1476 nm, respectively.

**Figure 6:**
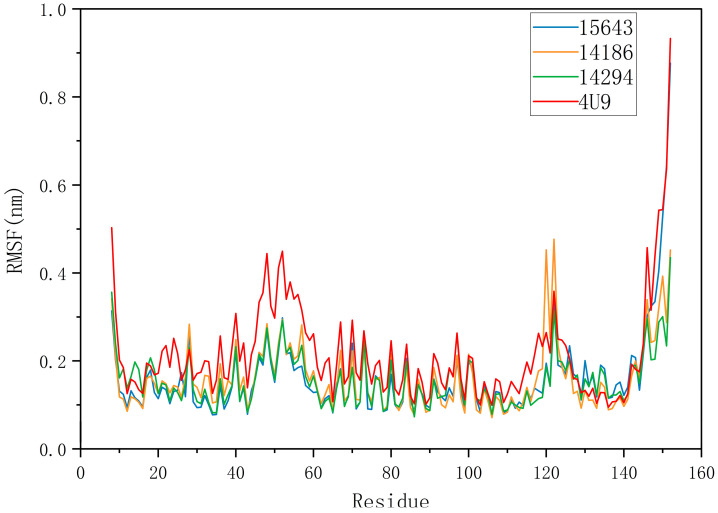
Root mean square fluctuation (RMSF) diagram for protein with positive compound 4U9 and three candidate compounds complexes.

The RMSF of a protein residue represents the root mean square shift of the residue in the protein conformation, which reflects the degree of freedom of the atom. As shown in Figure 6, RMSFs of the three compounds ranged from 0.0731 nm to 0.8764 nm. In general, the overall motion trend of the three compounds was consistent. The RMSF of 14186 fluctuated at residues 118–125 but then stabilized. RMSFs of 4U9 and 15643 appeared to peak at the end of the simulation, suggesting that they may have similar binding patterns. RMSD and RMSF analysis showed that 15643 had good binding stability with proteins, while 14294 and 14186 had ordinary binding stability with proteins. Therefore, compound 15643 is considered to have better binding stability.

#### 2.5.2. Hydrogen Bond Analysis

**Figure 7:**
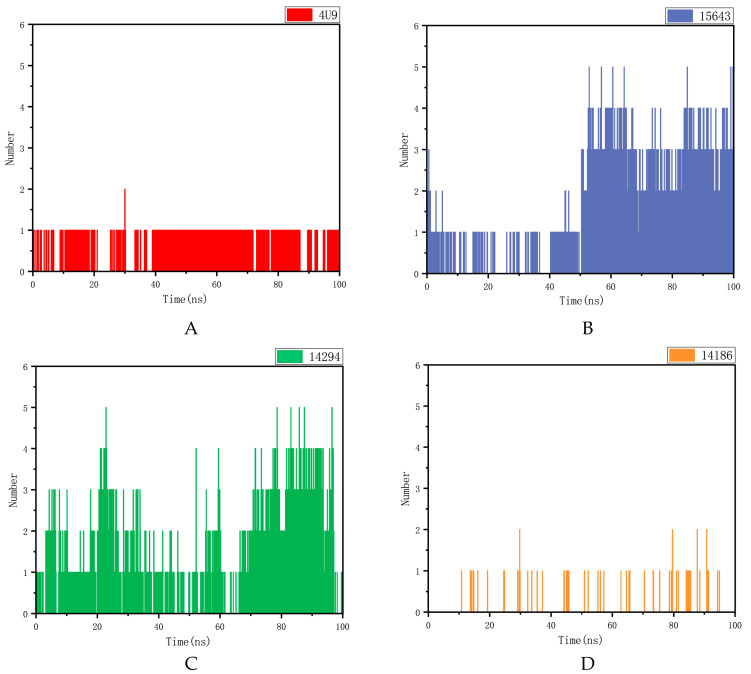
Hydrogen bond analysis of 4U9 and three compounds in the protein fitting process. (**A**) Positive compound 4U9 with protein. (**B**) Compound 15643 with protein. (**C**) Compound 14294 with protein. (**D**) Compound 14186 with protein.

The hydrogen bond between the protein and the ligand is an important factor in keeping the molecule within the active site cavity. As shown in Figure 7, compounds 14186 and mPGES-1 proprotein ligand complexes exhibited too few hydrogen bonds throughout the simulation. Compound 15643 also had fewer hydrogen bonds in the first 50 ns, while it formed more hydrogen bonds in the last 50 ns. Compound 14294 had fewer hydrogen bonds at 30 to 70 ns and more hydrogen bonds at 70 to 95 ns. As can be seen from these figures, compounds 15643 and 14294 formed more hydrogen bonds with mPGES1 during the simulation. It proves that compound 15643 has better interactions with proteins.

#### 2.5.3. MM-PBSA Analysis

**Figure 8:**
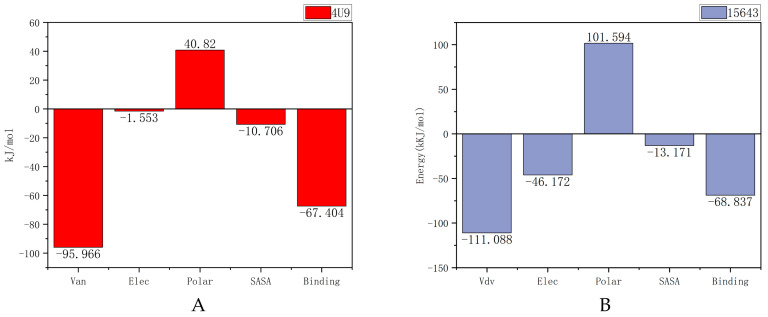
Binding energy analysis of 4U9 combined with protein and compound 15643. (**A**) Positive compound 4U9 with protein. (**B**) Compound 15643 with protein.

Poisson–Boltzmann surface area (MM-PBSA) is an effective and reliable method in molecular mechanics research, and MM-PBSA is used to calculate the free energy of compounds bound to their protein targets. The lower the free energy produced by the binding of proteins and compounds, the better the ligand binds to proteins. As shown in Figure 8, the free energies of compound 15643 and the original ligand are −68.837 kJ/mol and −67.404 kJ/mol, respectively, which are also mainly contributed by van der Waals forces. Obviously, 15643 and 14294 have binding free energies similar to or lower than 4U9.

The MM/GBSA for the complex system was modeled from the corresponding 100 ns (Table 3). The analysis of the contribution of each energy component showed that the screened compound 15643 could maintain a longer interaction with the protein than the original ligand.

## 3. Discussion

Makoto Murakami first proposed prostaglandin E synthase as a novel drug target for inflammation and cancer. In particular, gene targeting studies of mPGES-1 indicate that this enzyme represents a new target for anti-inflammatory and anti-cancer drugs [29]. In the following year, AbdulHameed MD, Hamza A et al. published a study on the binding of human microsomal prostaglandin E synthase-1 (mPGES-1) to inhibitors and its quantitative structure–activity correlation [30]. Subsequently, researchers continued to explore the development of mPGES-1 inhibitors, and several mPGES-1 inhibitors were reported between 2008 and 2023. Currently, Zaloglanstat (ISC-27864), an mPGES-1 inhibitor developed by Di Micco, S et al., which can be used to study asthma, osteoarthritis, rheumatoid arthritis, acute or chronic pain, and neurodegenerative diseases, has completed a Phase II clinical trial [31]. To date, researchers have discovered a range of mPGES-1 inhibitors with different chemical structures, such as MF63 antipyretic and analgesic effects of [2-(6-chloro-1*H*-phenanthro-[9,10-d]imidazol-2-yl)isophthalonitrile] in an inflammatory animal model [32]; the effective and selective dioxane-fused mPGES-1 inhibitor tricyclic benz [d] imidazole derivatives [33], oxadiazole thione-benzimidazole derivatives [34], and PF-4693627, mPGES-1 inhibitors that can be used in studies of osteoarthritis and rheumatoid arthritis [35]; etc. The discovery of these inhibitors has enriched the selection and research of mPGES-1 inhibitors. However, despite a long history of research on mPGES-1 inhibitors, there are currently no commercially available inhibitors of mPGES-1. With the deepening of Chinese medicine research, the use of Chinese medicine is more and more closely related to the field of drug discovery and molecular biology. Considering that small molecules of traditional Chinese medicine have become one of the important sources of rich therapeutic drugs, the purpose of our study was to screen mPGES-1 inhibitors from medicines collected and integrated from the Chinese medicine database.

The rapid development of computer technology has accelerated the development of drugs, and computer-aided design (CADD) has been widely used to rapidly screen out molecules with excellent targeted binding potential from large compound databases. In this study, the structure of the molecule was based on its virtual screening. We collected a batch of compounds containing IC50 values and certain inhibitory effects on mPGES-1 by reviewing the literature as a data set. Maestro was used to construct pharmacophore models based on the common pharmacophore characteristics of multiple ligands, and the constructed pharmacophore models were verified. The validated pharmacophore model was used in the first step of virtual screening, and only compounds meeting four of the five pharmacophore characteristics were retained.

As one of the computational tools for drug design and development, the docking method can predict the affinity of direct binding modes of compounds bound within protein binding sites and generate an energy score function for evaluating ligand posture by using corresponding algorithms to guide the selection of the screening results [36]. In this study, compounds screened using pharmacophores were butt screened successively for HTVS, SP, and XP, respectively, in order to improve the accuracy of butt screening. After analyzing the docking results, compounds in the top 20% of the docking scores were selected for the preliminary binding energy calculation of MMGBSA, which avoided the inaccuracy caused by relying solely on the docking scores. Finally, 10 compounds with better butt scores and MMGBSA binding free energy scores than the reference ligand 4U9 were selected for ADMET analysis. Only three of the ten compounds, compound 14294, compound 15643, and compound 14186, were in line with Lipinski’s five rules and had the lowest toxicity levels. The ADMET results of these four compounds were superior to those of the reference ligand 4U9, so they were reserved for kinetic studies.

Molecular dynamics studies are used to reflect the stability of compounds binding to proteins [37]. In this study, the crystal structure of mPGES-1 was respectively compared with three compounds of research value and the reference ligand 4U9 for 100 ns simulation locus, and RMSD, RMSF, and hydrogen bond analysis were performed. Compared with 4U9, compound 15643 had the property of more stable interaction with protein, so the other two compounds were removed. The binding energies between the protein and compounds 15643 and 4U9 were calculated and compared. The results showed that compound 15643 had better binding effect than 4U9.

Although the results of our study are scientific, there are still some shortcomings. The number of compounds in the Chinese medicine database selected for this study is 20,011, which is not enough, and the opportunity to screen better Chinese medicine compound inhibitors targeting mPGES-1 may be missed. In addition, we have not purchased the screened compounds for experimental verification, which does not make the accuracy of our research results 100% sure. At the same time, we also encountered challenges in the research process. In the screening process of the TCM database, the pharmacophore model construction had certain requirements regarding the number of compounds in the training set and the validation set, and more molecules with certain activities should be sought, to the degree possible, for the model construction and verification.

In conclusion, we identified an mPGES-1 inhibitor, compound 15643, from the traditional Chinese medicine database through multi-step virtual screening. This result provides a new reference for the development of mPGES-1 inhibitors.

## 4. Materials and Methods

### 4.1. Protein Structure

Suitable protein structures were selected from the protein database (https://www.rcsb.org/, accessed on 2 May 2022) for structure-based calculation to identify mPGES-1 inhibitors. The PDB ID of the selected protein was 4YL3. It is derived from the human protein structure with A conformational resolution of 1.41 A and complete protein conformational sequence, and the protein structure is complex with ligand (4U9), so it was selected as the object of study [38]. The complex structures of 4U9 and mPGES-1 were adjusted and optimized using the Protein Preparation module with the help of the Maestro visualization window of Schrodinger’s drug screening design and molecular simulation software. The 4U9 ligand binding site within the 3D receptor structure was designated as the binding region of the screening ligand, and a grid was created to surround the region.

### 4.2. Data Set

In this study, a database of traditional Chinese medicine compounds was selected as the screening object, and there were 20,011 compounds in the database. In order to ensure that the selected compounds have better drug utilization properties, the Maestro Ligprep module was used to optimize the conformation and energy settings of traditional Chinese medicine compounds. The LigPrep panel is used to set up and start configuration preparation calculations [39]. Our goal with LigPrep was to take the 2D structure and produce the corresponding low-energy 3D structure that Glide and programs can use. We used Epic to generate possible ionized states at pH 7.0 ± 2.0, resulting in tautomers, forming up to 32 stereoisomers per ligand, determining chirality from 3D coordinates, and producing low energy rings. Then, the OPLS3e force field was used to minimize conformation and energy [40]. Finally, more than 400,000 databases of TCM compounds in different conformations were generated for screening studies.

### 4.3. Pharmacophore Analysis

Pharmacophore modeling is a widely used method for calculating the chemical characteristics of the geometry of active sites and the spatial arrangement of ligand substituents in 3D space [41]. A number of known active mPGES-1 inhibitors were collected as data sets by reviewing extensive literature [38,42,43,44,45,46,47]. These were used to construct and validate the pharmacophore model. The Maestro program was used to carry out pharmacophore models based on ligand structure. After research and analysis, the model with the highest score and five pharmacophore characteristics (AAHNR) was selected. In addition, a total of 61 compounds containing IC50 values and that were active against mPGES-1 were collected from the literature search and compound library as the training set and verification set. Eighteen of them were training sets, and the rest were verification sets. The reliability of the model was evaluated from the AUC value of the ROC, which was between 0.5 and 1. The larger the value of AUC, the more likely the current classification algorithm will rank the positive samples before the negative samples; that is, the better the classification can potentially be [48]. The AUC value can be used to determine whether the model can be used for a pharmacophore based virtual screening.

After determining the selected pharmacophore model (AAHNR), the Maestro program was used to conduct the first virtual screening of compounds in the Chinese drug bank, namely the virtual screening based on the pharmacophore model [49]. The screening conditions were set as satisfying at least four of the five pharmacophore characteristics, and finally 270,000 compounds meeting the conditions were screened out from the Chinese drug bank.

### 4.4. Molecular Docking

After pharmacophore-based screening, compounds with high pharmacophore evaluation scores (truncation value set as 1) were selected for docking analysis. The Maestro tool was used to interconnect them with mPGES-1 for HTVS, SP, and XP in turn [50], aiming to gradually improve the accuracy of the interconnecting process. The compounds in the top 20% of the XP interconnecting score were selected for preliminary calculation of MMGBSA binding energy [51] and compared with 4U9. We selected 10 compounds with higher Glide scores and MMGBSA scores than 4U9 for further research.

### 4.5. ADMET

ADMET is a subject that quantitatively studies the process (absorption, distribution, metabolism, and excretion) of drugs in the biological body and describes the dynamic law of drugs in the body. It has become an important part of drug preclinical research and clinical research [27]. The SwissADME server (http://www.swissadme.ch/, accessed on 23 September 2022) was used to evaluate elected compounds after molecular docking ligands. The SMILES format was imported into the server, and the relevant parameters such as pharmacokinetic characteristics and drug solubility were combined to further select the compounds for the next operation.

### 4.6. Dynamic Simulation

Molecular dynamics simulations (MDs) were performed to evaluate the binding stability to proteins of the three candidate molecules. Before MDs were run, the simulation system was built using the GROMACS 2019.1 software package (sourced by Mark Abraham et al., Uppsala University, Stockholm University, and KTH Royal Institute of Technology, Stockholm, Sweden) [37]. The topological system of proteins was constructed by AMBER99SB-ILDN force field [52]. The Bio2byte web server (https://www.bio2byte.be/, accessed on 11 February 2023) and the GAFF force field were used to generate the molecular topology file [37,53]. In the simulation system, the cube box and TIP3P water model with radius of 1.2 nm were selected to define periodic boundary conditions (PBC) [54]. In addition, 8 chloride ions were added to each system to ensure the electrical neutrality of the simulated system; this step was calculated and completed using GROMACS. At the simulated temperature of 300 K, the system energy was minimized in 50,000 steps. In order to maintain the pressure and temperature of the system, the two simulation systems were balanced using position-constrained MD simulation at 300 K, lasting 100 ps. Finally, we ran a MD simulation with a duration of 100 ns. Through MD simulation, the root mean square deviations (RMSDs) and root mean square fluctuations (RMSFs) of atomic position were analyzed by extracting trajectory coordinates. In addition, the intermolecular H bond interaction between mPGES-1 and the ligand was extracted using the gmx-H bond analysis tool.

In order to calculate the free energy of receptor–ligand binding, this study adopted the molecular mechanics-Poisson–Boltzmann surface area (MM-PBSA) method, which was implemented using the GROMACS built-in tool g_mmpbsa [55]. The basic principle is to calculate the difference between the bound and unbound free energies of two solvated molecules or to compare the free energies of different solvated conformations of the same molecule. The combined free energy of recombination is calculated according to the following formula. $G_{\mathrm{complex}}$ Gcomplex represents the free energy of the protein–ligand complex, $G_{\mathrm{protein}}$ represents the free energy of the protein in the solvent, and $G_{\mathrm{ligand}}$ represents the free energy of the ligand in the solvent.

$$G_{\mathrm{binding}}=G_{\mathrm{complex}}-\left(G_{\mathrm{protein}}+G_{\mathrm{ligand}}\right)$$

The free energy of the protein–ligand complex is represented by the $G_{\mathrm{complex}}$, where the $G_{\mathrm{protein}}$ represents the free energy of the protein in the solvent, and $G_{\mathrm{ligand}}$ represents the free energy of the ligand in the solvent.

## 5. Conclusions

mPGES-1 plays an important role in inflammatory response and anti-tumor drug development. In this study, pharmacophore models were established based on the common pharmacodynamic characteristics of multiple ligands, and virtual screening was conducted based on the crystal structure of mPGES-1 and combined small-molecule inhibitor 4U9. A novel mPGES-1 inhibitor compound 15643 was identified by ADMET, dynamic simulation, and multi-step virtual screening. Compared with compound 4U9, compound 15643, which was finally screened in this study, had similar binding ability to the protein mPGES-1, and ADMET analysis showed that it had better pharmaceutical potential. In addition, compound 15643 has a more stable binding energy. Therefore, we can assume that compound 15643 has the potential to be an inhibitor of mPGES-1 in this study. This potential small-molecule inhibitor provides a new direction for the development of anti-cancer and anti-inflammatory drugs. The small molecule can be further evaluated via different laboratory-based experimental techniques to help determine the activity of the compound and provide reference values for the study of novel mPGES-1 inhibitors.

## Figures and Tables

**Table 1:** The ten compounds with the best Glide scores and MMGBSA scores compared.

Ligand	SP Docking Score (kcal/mol)	XP Docking Score (kcal/mol)	MMGBSA_dG_Bind
4U9	−3.291	−0.374	−49.058
Compound 13295	−3.355	−5.806	−51.275
Compound 14294	−3.140	−5.558	−57.754
Compound 14186	−3.129	−5.202	−49.202
Compound 9838	−4.184	−6.185	−49.225
Compound 15643	−3.197	−4.664	−55.336
Compound 12340	−3.520	−4.007	−55.425
Compound 13836	−3.404	−4.696	−57.291
Compound 13581	−3.464	−2.227	−54.882
Compound 12553	−3.474	−3.984	−50.575
Compound 13106	−3.782	−4.692	−50.208

**Table 2:** ADMET analysis of 4U9 and three selected compounds.

	4U9	15643	14186	14294
Chemical formula	C23H11BrCIF4N3	C21H18O12	C21H20O10	C19H14O12
Molecular weight	520.7	462.36	432.38	434.31
Hydrogen bond acceptors	6	12	10	12
Hydrogen bond donors	1	5	6	5
Water solubility (Log S)	−7.82	−2.95	−3.18	−2.25
Lipophilicity (Log P)	8.76	0.46	0.38	−0.04
Gastrointestinal absorption	Low	Low	Low	Low
Skin permeation (Log Kp (cm/s))	−4.5	−9.03	−8.33	−9.1
BBB permeation	NO	NO	NO	NO
P-glycoprotein substrate	YES	NO	NO	NO
Abbott bioavailability score	0.17	0.55	0.55	0.55
Toxicity of hepatotoxicity	Active	Inactive	Inactive	Inactive
Carcinogenicity	Inactive	Inactive	Inactive	Active
Immunotoxicity	Active	Active	Inactive	Active
Mutagenicity	Inactive	Active	Inactive	Active
Cytotoxicity	Inactive	Inactive	Inactive	Inactive
Predicted LD50 (mg/kg)	2000	5000	2500	1100
Predicted toxicity class	4	5	5	4

**Table 3:** Binding energy analysis of compound 4U9 and compound 15643 combined with proteins.

Criteria	Title 2	Title 3
Van der Waal energy (kJ/mol)	−95.966	−111.088
Electrostatic energy (kJ/mol)	−1.533	−46.172
Polar solvation energy (kJ/mol)	40.820	101.594
SASA energy (kJ/mol)	−10.706	−13.171
Binding energy (kJ/mol)	−67.404	−68.837

## Data Availability

The data used to support the findings of this study are included within the article.

## Supplementary Materials

The following supporting information can be downloaded at: https://www.mdpi.com/article/10.3390/molecules28166059/s1, Figure S1: Relationship between PG and inflammation and cancer; Figure S2: The virtual screening workflow (VSW) used in this study was used to identify targeted mPGES-1 inhibitors; Figure S3: Compound structure and IC50 values for pharmacophore modeling.

## References

[1] Ricciotti E., FitzGerald G.A. (2011). Prostaglandins and inflammation. Arter. Thromb. Vasc. Biol..

[2] Takeuchi K. (2011). [Development and repair of NSAIDs-induced small intestinal lesions: Relation to COX isozymes and EP receptor subtypes]. Nihon. Rinsho..

[3] Bogdan D., Falcone J., Kanjiya M.P., Park S.H., Carbonetti G., Studholme K., Gomez M., Lu Y., Elmes M.W., Smietalo N. (2018). Fatty acid-binding protein 5 controls microsomal prostaglandin E synthase 1 (mPGES-1) induction during inflammation. J. Biol. Chem..

[4] Takahashi R., Amano H., Ito Y., Eshima K., Satoh T., Iwamura M., Nakamura M., Kitasato H., Uematsu S., Raouf J. (2020). Microsomal prostaglandin E synthase-1 promotes lung metastasis via SDF-1/CXCR4-mediated recruitment of CD11b(+)Gr1(+)MDSCs from bone marrow. Biomed. Pharmacother..

[5] van Rees B.P., Sivula A., Thorén S., Yokozaki H., Jakobsson P.J., Offerhaus G.J., Ristimäki A. (2003). Expression of microsomal prostaglandin E synthase-1 in intestinal type gastric adenocarcinoma and in gastric cancer cell lines. Int. J. Cancer.

[6] Yoshimatsu K., Golijanin D., Paty P.B., Soslow R.A., Jakobsson P.J., DeLellis R.A., Subbaramaiah K., Dannenberg A.J. (2001). Inducible microsomal prostaglandin E synthase is overexpressed in colorectal adenomas and cancer. Clin. Cancer Res..

[7] Xu L.W., Qian M., Jia R.P., Xu Z., Wu J.P., Li W.C., Huang W.B., Chen X.G. (2012). Expression and significance of microsomal prostaglandin synthase-1 (mPGES-1) and Beclin-1 in the development of prostate cancer. Asian Pac. J. Cancer Prev..

[8] Mehrotra S., Morimiya A., Agarwal B., Konger R., Badve S. (2006). Microsomal prostaglandin E2 synthase-1 in breast cancer: A potential target for therapy. J. Pathol..

[9] Herfs M., Herman L., Hubert P., Minner F., Arafa M., Roncarati P., Henrotin Y., Boniver J., Delvenne P. (2009). High expression of PGE2 enzymatic pathways in cervical (pre)neoplastic lesions and functional consequences for antigen-presenting cells. Cancer Immunol. Immunother..

[10] Hasan S., Satake M., Dawson D.W., Funahashi H., Angst E., Go V.L., Reber H.A., Hines O.J., Eibl G. (2008). Expression analysis of the prostaglandin E2 production pathway in human pancreatic cancers. Pancreas.

[11] Kim S.H., Roszik J., Cho S.N., Ogata D., Milton D.R., Peng W., Menter D.G., Ekmekcioglu S., Grimm E.A. (2019). The COX2 Effector Microsomal PGE2 Synthase 1 is a Regulator of Immunosuppression in Cutaneous Melanoma. Clin. Cancer Res..

[12] Leoncini E., Ricciardi W., Cadoni G., Arzani D., Petrelli L., Paludetti G., Brennan P., Luce D., Stucker I., Matsuo K. (2014). Adult height and head and neck cancer: A pooled analysis within the INHANCE Consortium. Eur. J. Epidemiol..

[13] Omi Y., Shibata N., Okamoto T., Obara T., Kobayashi M. (2009). Immunohistochemical demonstration of membrane-bound prostaglandin E2 synthase-1 in papillary thyroid carcinoma. Acta Histochem. Cytochem..

[14] Mattila S., Tuominen H., Koivukangas J., Stenbäck F. (2009). The terminal prostaglandin synthases mPGES-1, mPGES-2, and cPGES are all overexpressed in human gliomas. Neuropathology.

[15] Chini M.G., Giordano A., Potenza M., Terracciano S., Fischer K., Vaccaro M.C., Colarusso E., Bruno I., Riccio R., Koeberle A. (2020). Targeting mPGES-1 by a Combinatorial Approach: Identification of the Aminobenzothiazole Scaffold to Suppress PGE(2) Levels. ACS Med. Chem Lett..

[16] Korotkova M., Jakobsson P.J. (2014). Characterization of microsomal prostaglandin E synthase 1 inhibitors. Basic Clin Pharmacol. Toxicol..

[17] Sun Y. (2014). The role of Chinese medicine in clinical oncology. Chin. J. Integr. Med..

[18] Huang Y., Cai T., Xia X., Cai Y., Wu X.Y. (2016). Research Advances in the Intervention of Inflammation and Cancer by Active Ingredients of Traditional Chinese Medicine. J. Pharm. Pharm. Sci..

[19] Kumar Bhardwaj V., Purohit R., Kumar S. (2021). Himalayan bioactive molecules as potential entry inhibitors for the human immunodeficiency virus. Food Chem..

[20] Tanwar G., Mazumder A.G., Bhardwaj V., Kumari S., Bharti R., Yamini, Singh D., Das P., Purohit R. (2019). Target identification, screening and in vivo evaluation of pyrrolone-fused benzosuberene compounds against human epilepsy using Zebrafish model of pentylenetetrazol-induced seizures. Sci. Rep..

[21] Yu W., MacKerell A.D. (2017). Computer-Aided Drug Design Methods. Methods Mol. Biol..

[22] Sun H. (2008). Pharmacophore-based virtual screening. Curr. Med. Chem..

[23] Luo L., Zhong A., Wang Q., Zheng T. (2021). Structure-Based Pharmacophore Modeling, Virtual Screening, Molecular Docking, ADMET, and Molecular Dynamics (MD) Simulation of Potential Inhibitors of PD-L1 from the Library of Marine Natural Products. Mar. Drugs.

[24] Tai W., Lu T., Yuan H., Wang F., Liu H., Lu S., Leng Y., Zhang W., Jiang Y., Chen Y. (2012). Pharmacophore modeling and virtual screening studies to identify new c-Met inhibitors. J. Mol. Model..

[25] Martínez Pérez J.A., Pérez Martin P.S. (2023). [ROC curve]. Semergen.

[26] Friesner R.A., Banks J.L., Murphy R.B., Halgren T.A., Klicic J.J., Mainz D.T., Repasky M.P., Knoll E.H., Shelley M., Perry J.K. (2004). Glide: A new approach for rapid, accurate docking and scoring. 1. Method and assessment of docking accuracy. J. Med. Chem..

[27] Ferreira L.L.G., Andricopulo A.D. (2019). ADMET modeling approaches in drug discovery. Drug Discov. Today.

[28] Daina A., Michielin O., Zoete V. (2017). SwissADME: A free web tool to evaluate pharmacokinetics, drug-likeness and medicinal chemistry friendliness of small molecules. Sci. Rep..

[29] Murakami M., Kudo I. (2006). Prostaglandin E synthase: A novel drug target for inflammation and cancer. Curr. Pharm. Des..

[30] AbdulHameed M.D., Hamza A., Liu J., Huang X., Zhan C.G. (2008). Human microsomal prostaglandin E synthase-1 (mPGES-1) binding with inhibitors and the quantitative structure-activity correlation. J. Chem. Inf. Model..

[31] Di Micco S., Terracciano S., Ruggiero D., Potenza M., Vaccaro M.C., Fischer K., Werz O., Bruno I., Bifulco G. (2021). Identification of 2-(thiophen-2-yl)acetic Acid-Based Lead Compound for mPGES-1 Inhibition. Front. Chem..

[32] Xu D., Rowland S.E., Clark P., Giroux A., Côté B., Guiral S., Salem M., Ducharme Y., Friesen R.W., Méthot N. (2008). MF63 [2-(6-chloro-1H-phenanthro[9,10-d]imidazol-2-yl)-isophthalonitrile], a selective microsomal prostaglandin E synthase-1 inhibitor, relieves pyresis and pain in preclinical models of inflammation. J. Pharmacol. Exp. Ther..

[33] Muthukaman N., Deshmukh S., Sarode N., Tondlekar S., Tambe M., Pisal D., Shaikh M., Kattige V.G., Honnegowda S., Karande V. (2016). Discovery of 2-((2-chloro-6-fluorophenyl)amino)-N-(3-fluoro-5-(trifluoromethyl)phenyl)-1-methyl-7,8-dihydro-1H-[1,4]dioxino[2′,3′:3,4]benzo[1,2-d]imidazole-5-carboxamide as potent, selective and efficacious microsomal prostaglandin E(2) synthase-1 (mPGES-1) inhibitor. Bioorg. Med. Chem. Lett..

[34] Ergül A.G., Maz T.G., Kretzer C., Olğaç A., Jordan P.M., Çalışkan B., Werz O., Banoglu E. (2022). Novel potent benzimidazole-based microsomal prostaglandin E(2) synthase-1 (mPGES-1) inhibitors derived from BRP-201 that also inhibit leukotriene C(4) synthase. Eur. J. Med. Chem..

[35] Arhancet G.B., Walker D.P., Metz S., Fobian Y.M., Heasley S.E., Carter J.S., Springer J.R., Jones D.E., Hayes M.J., Shaffer A.F. (2013). Discovery and SAR of PF-4693627, a potent, selective and orally bioavailable mPGES-1 inhibitor for the potential treatment of inflammation. Bioorg. Med. Chem. Lett..

[36] Guedes I.A., de Magalhães C.S., Dardenne L.E. (2014). Receptor-ligand molecular docking. Biophys. Rev..

[37] Van Der Spoel D., Lindahl E., Hess B., Groenhof G., Mark A.E., Berendsen H.J. (2005). GROMACS: Fast, flexible, and free. J. Comput. Chem..

[38] Luz J.G., Antonysamy S., Kuklish S.L., Condon B., Lee M.R., Allison D., Yu X.P., Chandrasekhar S., Backer R., Zhang A. (2015). Crystal Structures of mPGES-1 Inhibitor Complexes Form a Basis for the Rational Design of Potent Analgesic and Anti-Inflammatory Therapeutics. J. Med. Chem..

[39] Sheikh I.A., Jiffri E.H., Ashraf G.M., Kamal M.A., Beg M.A. (2018). Structural studies on inhibitory mechanisms of antibiotic, corticosteroid and catecholamine molecules on lactoperoxidase. Life Sci..

[40] Babaoglu Z.Y., Kilic D. (2023). Virtual screening, molecular simulations and bioassays: Discovering novel microsomal prostaglandin E Synthase-1 (mPGES-1) inhibitors. Comput. Biol. Med..

[41] Yadav D.K., Kumar S., Teli M.K., Kim M.H. (2020). Ligand-based pharmacophore modeling and docking studies on vitamin D receptor inhibitors. J. Cell Biochem..

[42] Schiffler M.A., Antonysamy S., Bhattachar S.N., Campanale K.M., Chandrasekhar S., Condon B., Desai P.V., Fisher M.J., Groshong C., Harvey A. (2016). Discovery and Characterization of 2-Acylaminoimidazole Microsomal Prostaglandin E Synthase-1 Inhibitors. J. Med. Chem..

[43] Shekfeh S., Caliskan B., Fischer K., Yalcin T., Garscha U., Werz O., Banoglu E. (2019). A Multi-step Virtual Screening Protocol for the Identification of Novel Non-acidic Microsomal Prostaglandin E(2) Synthase-1 (mPGES-1) Inhibitors. ChemMedChem.

[44] Misra S., Saini M., Ojha H., Sharma D., Sharma K. (2017). Pharmacophore modelling, atom-based 3D-QSAR generation and virtual screening of molecules projected for mPGES-1 inhibitory activity. SAR QSAR Environ. Res..

[45] Lauro G., Cantone V., Potenza M., Fischer K., Koeberle A., Werz O., Riccio R., Bifulco G. (2018). Discovery of 3-hydroxy-3-pyrrolin-2-one-based mPGES-1 inhibitors using a multi-step virtual screening protocol. Medchemcomm.

[46] Psarra A., Nikolaou A., Kokotou M.G., Limnios D., Kokotos G. (2017). Microsomal prostaglandin E(2) synthase-1 inhibitors: A patent review. Expert Opin. Ther. Pat..

[47] Waltenberger B., Wiechmann K., Bauer J., Markt P., Noha S.M., Wolber G., Rollinger J.M., Werz O., Schuster D., Stuppner H. (2011). Pharmacophore modeling and virtual screening for novel acidic inhibitors of microsomal prostaglandin E(2) synthase-1 (mPGES-1). J. Med. Chem..

[48] Muschelli J. (2020). ROC and AUC with a Binary Predictor: A Potentially Misleading Metric. J. Classif..

[49] Löwer M., Proschak E. (2011). Structure-Based Pharmacophores for Virtual Screening. Mol. Inform..

[50] Alogheli H., Olanders G., Schaal W., Brandt P., Karlén A. (2017). Docking of Macrocycles: Comparing Rigid and Flexible Docking in Glide. J. Chem. Inf. Model..

[51] Bathula R., Lanka G., Muddagoni N., Dasari M., Nakkala S., Bhargavi M., Somadi G., Sivan S.K., Rajender Potlapally S. (2020). Identification of potential Aurora kinase-C protein inhibitors: An amalgamation of energy minimization, virtual screening, prime MMGBSA and AutoDock. J. Biomol. Struct. Dyn..

[52] Wang J., Wolf R.M., Caldwell J.W., Kollman P.A., Case D.A. (2004). Development and testing of a general amber force field. J. Comput. Chem..

[53] Hornak V., Abel R., Okur A., Strockbine B., Roitberg A., Simmerling C. (2006). Comparison of multiple Amber force fields and development of improved protein backbone parameters. Proteins.

[54] Harrach M.F., Drossel B. (2014). Structure and dynamics of TIP3P, TIP4P, and TIP5P water near smooth and atomistic walls of different hydroaffinity. J. Chem. Phys..

[55] Kumari R., Kumar R., Lynn A. (2014). g_mmpbsa--a GROMACS tool for high-throughput MM-PBSA calculations. J. Chem. Inf. Model.

