# Supplementary material for: mPGES-1 Inhibitor Discovery Based on Computer-Aided Screening: Pharmacophore Models, Molecular Docking, ADMET, and MD Simulations
Source: Molecules. 2023 Aug 15;28(16):6059. doi: 10.3390/molecules28166059 (PMC10458489; doi:10.3390/molecules28166059)
Supplement: Supplementary file 1 [file molecules-2512089-supplementary.pdf › molecules-2512089-supplementary.pdf]

Dear Editor: In your comments returned this time, you mentioned the issue of supplementary materials. We have submitted the documents of supplementary materials when we submitted the manuscript last time, and now we submit them again to prevent any omissions. For The "Supplementary Materials:" part of the article, "The following supporting information can be downloaded at: www.mdpi.com/xxx/s1 ", we can't confirm the content for the time being, we can only submit the supplementary materials to you, please let you edit.

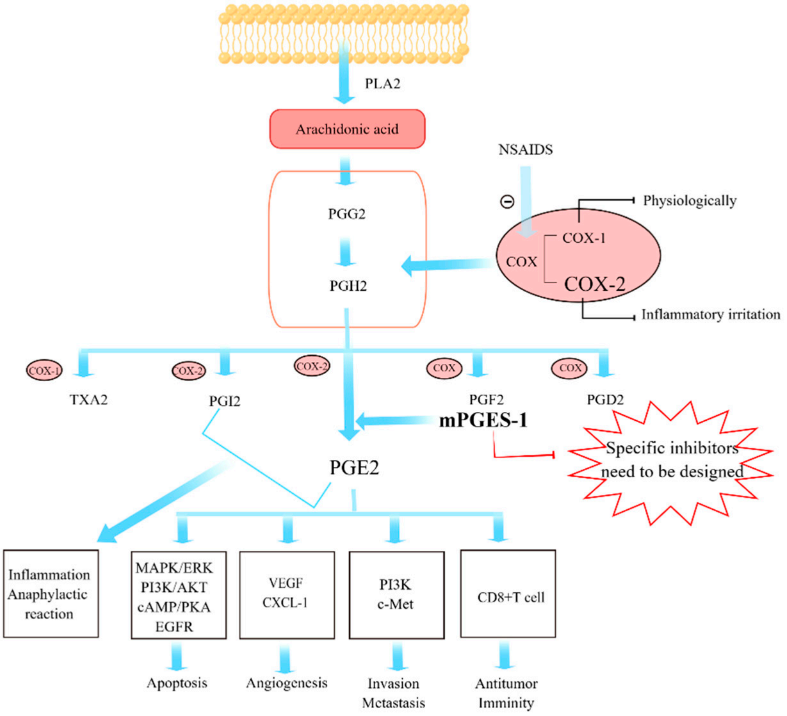

Figure S1. Relationship between PG and inflammation and cancer. The anti-inflammatory and antitumor effects of PGE2 can be selectively inhibited using mPGES-1 inhibitors.

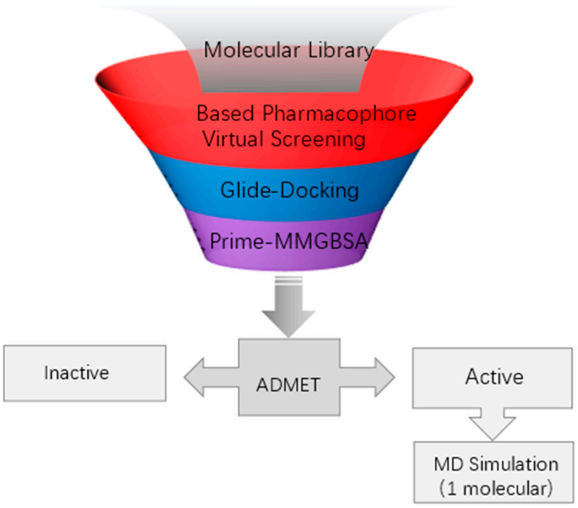

Figure S2. The virtual screening workflow (VSW) used in this study was used to identify targeted mPGES-1 inhibitors.

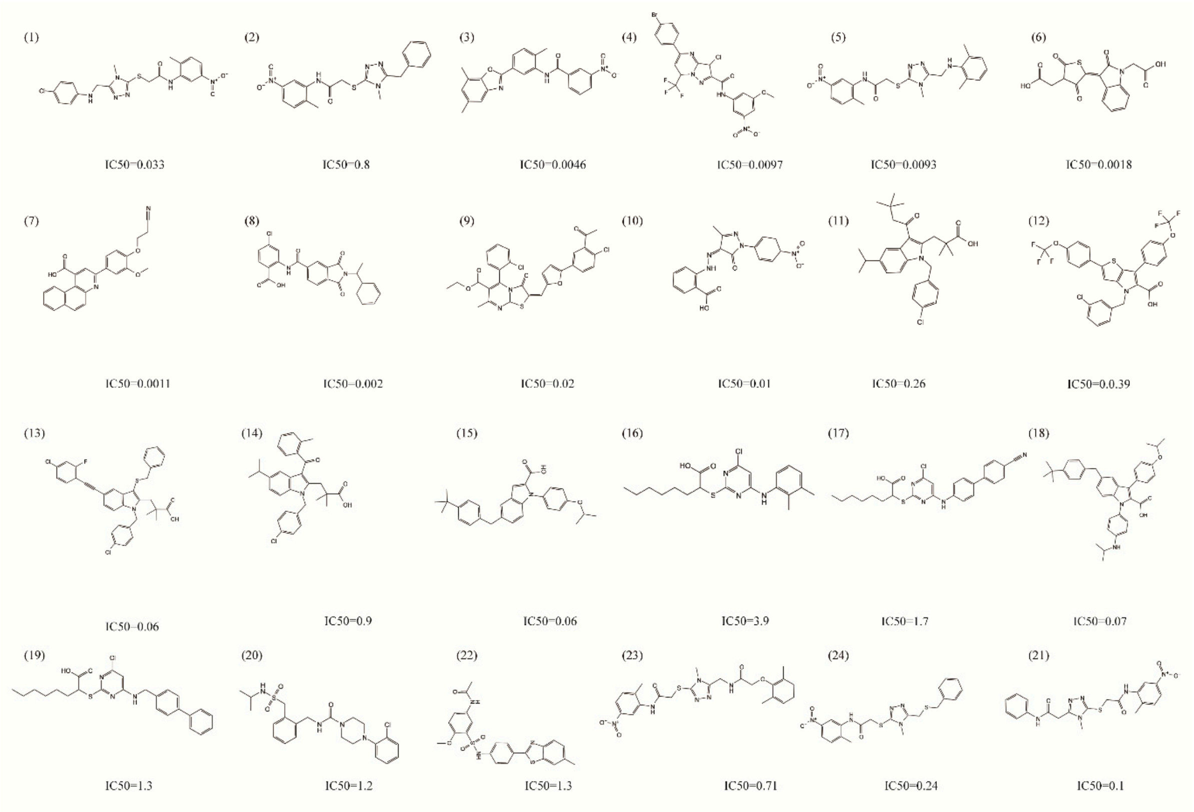

Figure S3. Compound structure and IC50 values for pharmacophore modeling
